# Mapping the nutritional value of diets across Europe according to the Nutri-Score front-of-pack label

**DOI:** 10.3389/fnut.2022.1080858

**Published:** 2023-01-13

**Authors:** Elly Mertens, José L. Peñalvo

**Affiliations:** Unit of Non-communicable Diseases, Department of Public Health, Institute of Tropical Medicine, Antwerp, Belgium

**Keywords:** Nutri-Score, nutrient profiling, Food Consumption Survey, Europe, diet quality

## Abstract

**Background:**

Front-of-pack labels, such as Nutri-Score, aim to offer clear information on the overall nutritional quality of foods and beverages to consumers, allowing them to make healthier food choices. Using the European Food Safety Authority (EFSA) Comprehensive European Food Consumption Database, the present study aims to map out European food consumption patterns by applying the Nutri-Score as a benchmark for nutritional value.

**Methods:**

Country-specific food consumption data, collected by multiple 24-h dietary recalls or food records available from EFSA, were linked to the Dutch Food Composition Database (NEVO). Foods and beverages consumed by adolescents (10–17 years), adults (18–64 years), and the elderly (65–74 years) were graded following the modified Food Standard Agency Nutrient Profiling System (FSAm-NPS) and classified according to Nutri-Score grading, from A to E. Subsequently, a dietary index score (FSAm-NPS-DI) was calculated for each country-specific diet by age-groups and sex as an energy-weighted mean of the FSAm-NPS score of all foods and beverages consumed, with lower scores for a diet of greater overall nutritional quality.

**Results:**

On average, the daily energy intake of adults across the European countries studied is distributed in 27.6% of A-, 12.9% of B-, 17% of C-, 30.0% of D-, and 12.5% of E-classified foods and beverages. This energy distribution, according to the Nutri-Score, corresponded to a median FSAm-NPS-DI score of 6.34 (interquartile range: 5.92, 7.19). For both adult males and females, Estonia reported the highest energy share from A-classified products, scoring the lowest on the FSAm-NPS-DI. On the other hand, Latvia reported the highest energy share from E-classified products, along with the highest FSAm-NPS-DI. Females and the elderly group reported, in general, a greater energy share from A- and a lower share from E-classified products, and had the lowest FSAm-NPS-DI scores. No sex-related difference was observed for adolescents whose share of energy was predominantly from A- and D-classified products, such as for adults and the elderly.

**Conclusion:**

Our analyses leveraging the secondary use of country-specific databases on dietary intakes found considerable variation in the nutritional value of European diets, with an overall agreement across all countries on a modestly healthier dietary profile for the elderly and among females.

## Introduction

Suboptimal diets are a leading risk factor for non-communicable diseases (NCDs), with an estimated 8 million deaths globally attributed to dietary risks in 2019 (1). In an effort to curb this burden, front-of-pack nutrition labeling is regarded as an internationally recognized strategy for nudging consumers toward healthy food choices (2–4). A front-of-pack label, providing easy-to-convey nutritional information about foods, has the potential to not only promote healthful purchasing behavior but also encourage food manufacturers to improve the nutrition profile of their products (5).

The Nutri-Score, a five color-coded front-of-pack labeling system, synthesizes the mandatory nutrition declaration, as often available on the back of the packaging, into five categories (ranging from A in dark green color, indicating greater nutritional quality, to E in dark red color, indicating lower nutritional quality) (6). The nutrient profiling system underlying the Nutri-Score was initially developed by the British Food Standards Agency (7), and after an update of the algorithm in 2017, the Nutri-Score has been subsequently adopted in France (October 2017) (8, 9), Spain (November 2018), Belgium (April 2019), Germany and Switzerland (September 2019), the Netherlands (November 2019), and Luxembourg (February 2020) as a voluntary tool for front-of-pack labeling, with the potential for improving public health nutrition.

Emerging evidence from experimental studies supports the effectiveness of the Nutri-Score in the European context, with reference to consumers' ability to correctly classify foods according to nutritional quality (10–12) as well as eliciting healthier food choices (11) and purchases (13) and lowering portion size selection of less healthy foods (14). In addition, when the nutritional profiles of foods underlying the Nutri-Score are applied to grading the quality of the overall diet of a population, individuals consuming a greater proportion of foods ranking low in nutritional quality have been observed to carry a higher risk of cardiovascular disease, as reported in the Supplementation en Vitamines et Mineraux Antioxydants (SU.VI.MAX) study (15) and the NutriNet-Santé (16) cohort, cancer as reported in the SU.VI.MAX study (17) and in the multinational European Prospective Investigation into Cancer and Nutrition (EPIC) cohort (18), and all-cause and disease-specific mortality in EPIC (19), Whitehall II (20), Estudio de Nutrición y Riesgo Cardiovascular en España (ENRICA) (21), and Seguimiento Universidad de Navarra (SUN) (22) cohorts. In regard to these consistent associations, monitoring the diets of the population from the perspective of overall nutritional quality—as provided, for example, by the Nutri-Score—appears to be important for public health, food policy planning, and addressing the increasing burden of NCD risk factors and would be particularly relevant for the development and implementation of strategies promoting healthy eating for all, given the urgent need to address health equity, as acknowledged by the World Health Organization (23, 24) and the European Union (23, 25). Still, before implementing a large-scale introduction of the Nutri-Score as a front-of-pack label, its effectiveness in improving food purchases toward healthier options needs to be measured in real-life supermarket settings with a complete assortment of products bearing the label (26). In recent years, public health policies and actions have increasingly acknowledged the potential of easy-to-read front-of-pack labels, which can offer a clear message on the overall nutritional quality of foods and beverages. In this line, the European Commission intents to propose a harmonized mandatory front-of-pack label at the EU level by the end of 2022, for guiding consumers toward healthier food choices (27). Although some EU countries have already introduced the voluntary use of the Nutri-Score and reported the nutritional quality of their diets according to the algorithm, a standardized assessment of the nutritional value of diets across Europe, including countries where the Nutri-Score has not been implemented, is lacking. Using EU members national dietary survey data compiled and standardized by the European Food Safety Authority (EFSA) Comprehensive European Food Consumption Database, this study aims to assess the nutritional value of diets across Europe using the Nutri-Score front-of-pack label and its underlying nutrient profiling system algorithm as a benchmark. This information aims to serve as the basis to study the use of the Nutri-Score as a potentially effective food policy before the introduction of a mandatory, science-based, and consumer-friendly nutrition label.

## Materials and methods

### Food consumption data

Country-level food consumption data estimated from individual-level national dietary surveys were obtained from the publicly available Comprehensive European Food Consumption Database, developed and maintained since 2011 by the European Food Safety Authority (EFSA) (28). The summary statistics of their food consumption data reported in grams/day (population mean intakes) and classified according to the sixth level of the “Exposure Hierarchy” of the comprehensive food classification and description system FoodEx2 (29–31) were retrieved, aggregated for adolescent (10–17 years), adult (18–64 years), and elderly (65–74 years) populations, stratified by sex. From the 25 European countries reporting survey dietary data to EFSA (in total, 69 dietary surveys), we selected the surveys for which food consumption data were available for at least 2 days, that is, 27 surveys in 19 countries for adolescents, 34 surveys in 22 countries for adults, and 25 surveys in 20 countries for the elderly (28).

### Linkage with the dutch food composition database (NEVO)

FoodEx2-coded food consumption data of EFSA were linked to food composition and corresponding nutritional information, using the the Dutch Food Composition Database version 2019 (Nederlands Voedingsstoffenbestand; NEVO) (32). This linkage was initiated in the Dutch Food Consumption Survey (2012–2016) (33), where the foods consumed were coded according to NEVO codes as well as FoodEx2 codes. Thereafter, we extended the classification to the remaining FoodEx2 codes available in the EFSA database using the NEVO code that most closely resembled the level-six description of the FoodEx2 “Exposure Hierarchy.” This linkage with NEVO allowed nutrient profiling and classifying foods according to the Nutri-Score front-of-pack label system.

### Computation of the nutrient profiling and the derived Nutri-Score

The Nutri-Score relies on a nutrient profiling system derived from the U.K. Food Standards Agency Nutrient Profiling System (FSA-NPS), initially developed to regulate television advertising to children (34). The profile system was later modified by the French High Council of Public Health (35), with regard to point allocations for beverages, cheese, and added fats for improved discrimination of nutritional quality within these food groups. Details of the modified Food Standards Agency Nutrient Profiling System (FSAm-NPS), including the derivation of the Nutri-Score, for each food and beverage have been published in detail elsewhere (7, 9, 35, 36). In brief, for each FoodEx2-coded food or beverage item included in the country-specific EFSA dietary database and linked with the NEVO, we calculated the FSAm-NPS based on the item composition (per 100 g or mL of content). Positive points (i.e., A points for the nutrients to be limited) were allocated, following the grid for point allocations for each item, energy (kJ), total sugar (g), saturated fatty acids (g or % of total lipids for fats and oils), and sodium (mg) content, and negative points (i.e., C points for the food groups and nutrients to be endorsed) for fruits, vegetables, nuts and legumes (%), fiber (g), and protein (g) content. The A points with a range of 0–10 for each and C points with a range of 0–5 for each were summed, and subsequently, the sum of C points was subtracted from the sum of A points to categorize the foods and beverages according to the Nutri-Score cutoffs.

The FSAm-NPS for each food or beverage is based on a unique discrete continuous scale ranging from −15 points (highest nutritional quality) to 40 points (lowest nutritional quality), and arithmetic energy-weighted means were aggregated to calculate a score at the diet level (FSAm-NPS-DI) using with the following equation (37):


(1) $$\begin{array}{l} FSAm-NPS-DI=\frac{\sum_{i=1}^{n}FSi^{*}Ei}{\sum_{i=1}^{n}Ei}\; \end{array}$$

where FSi represents the food score based on the FSAm-NPS of food or beverage i, Ei represents the energy intake from the item i, and n represents the number of items consumed. A higher FSAm-NPS-DI reflects a diet with high consumption of items ranked low in nutritional quality.

### Data analysis

For the most recent survey year for each country, the calculated FSAm-NPS algorithm was subsequently classified according to the Nutri-Score grading, from A to E. Applying the Nutri-Score to the diet, we expressed the nutritional value of the diet by the proportion of daily food consumption from foods and beverages classified as A, B, C, D, and E, as well as by the share of daily energy intake from A-, B-, C-, D-, and E-classified foods and beverages. The mean energy contribution of the food subgroups to the dietary energy share of the Nutri-Score categories was calculated in order to identify the top five contributors for each.

To facilitate the interpretation of the overall nutritional quality of the diet, we calculated the FSAm-NPS-DI for each survey included. For those countries with recurrent dietary surveys over time, we visualized the time changes in the FSAm-NPS-DI, provided that the same dietary assessment method was used for dietary data collections, that is, either the 24-h recall or food record for the recurrent dietary surveys.

## Results

### Nutritional value of European diets according to the Nutri-Score

**Figure 1:**
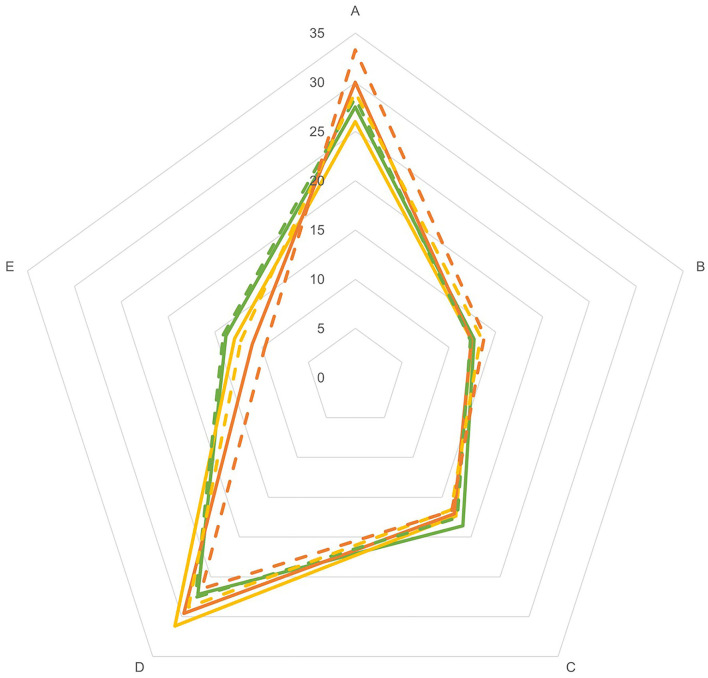
Nutritional value of European diets according to the Nutri-Score classification, expressed in the share (%) of dietary energy intake from foods and beverages classified as A, B, C, D, and E, stratified by age-group and sex. 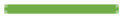 *Adolescent-Males;* 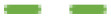 *Adolescent-Females;* 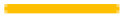 *Adult-Males;* 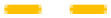 *Adult-Females;* 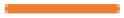 *Elderly-Males;* 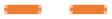 *Elderly-Females*.

Supplementary Tables 1–3 show the nutritional value of individuals' diet across European countries according to the Nutri-Score classification and the FSAm-NPS-DI, stratified by age-group and sex. On average in Europe, for adults, the classification of daily food and beverage consumption according to the Nutri-Score was 56.4% for A-, 11.8% for B-, 13.3% for C-, 12.3% for D-, and 6.2% for E-classified foods and beverages. These corresponded to a share of total energy intake from foods and beverages classified as 27.6% for A-, 12.9% for B-, 17.0% for C-, 30.0% for D-, and 12.5% for E-classified foods and beverages (Supplementary Table 2). A higher energy share from products classified as A and a lower energy share from E were observed for the elderly group and females, except for adolescents (Figure 1).

In adult males, the dietary share of energy intake from A-classified products ranged from 35.4% (Estonia) and 32.5% (Denmark) to 16.4% (Greece) and that from E ranged from 7.9% (Italy) to 19.6% (Latvia) (Supplementary Table 2). Similarly, in adults females, A-classified products ranged from 39.6% (Estonia) and 36.7% (Denmark) to 19.7% (Greece) and E from 6.6% (Italy) to 20.3% (Latvia). It is apparent from Supplementary Tables 1, 3 that similar to the adults' diet, the nutritional value according to the Nutri-Score of the diets of the adolescents and the elderly shows variability across the countries, and that, in general, similar countries were named the countries with a diet high vs. low in A- and E-classified products. Nevertheless, for both sexes, the energy share of A-classified products was also observed to be higher in Finland for adolescents, and in Portugal and Slovenia among the elderly, while it was lower for the elderly in France and Latvia (males only). A higher energy share of E-classified products was observed for Austria, Germany, and the United Kingdom in the diet of the adolescents and the elderly for both sexes but was lower for Portugal.

### Main food contributors to Nutri-Score A, B, C, D, and E ranks

There is a large variability in the nutritional composition of foods and beverages within a generic food group (Table 1). This is particularly evident for bread, breakfast cereals, fine bakery wares, dairy products, cheese, and meat, which were the top five contributors to multiple Nutri-Score ranks. Albeit with a large variability in their percentage contribution, most countries included similar food groups in their top five contributors of Nutri-Score rank A. The top five contributors for the lower ranks of the Nutri-Score, however, showed diverse food groups across countries and, to a lesser extent, between age-groups and sex. Nevertheless, in all diets studied, fats and oils were identified as one of the main contributors in the intake share of D-classified products, and in most of them, the top five of rank E included meat, fine bakery wares, chocolate, fruit juices, alcohol (adults and elderly only), soft drinks (adolescents only), and cheese.

**Table 1:** Main five food group contributors^a^ to the A, B, C, D, and E Nutri-Score ranks in Europe by age-groups and sex.

	**Adolescents**		**Adults**		**Elderly**	
	**Males**	**Females**	**Males**	**Females**	**Males**	**Females**
**N countries**	**21**	**21**	**22**	**22**	**20**	**20**
**Nutri-Score A**						
Cereals, pasta, rice	19 (8–42%)	17 (12–44%)	17 (8–35%)	16 (8–33%)	8 (3–28%)	8 (8–26%)
Bread	15 (9–45%)	15 (10–41%)	21 (11–47%)	21 (12–41%)	19 (15–62%)	20 (10–51%)
Potatoes	18 (5–20%)	20 (6–19%)	20 (5–19%)	16 (8–15%)	19 (4–19%)	18 (7–15%)
Fruit	20 (5–21%)	21 (9–24%)	20 (7–23%)	22 (10–27%)	20 (10–30%)	20 (14–29%)
Dairy products	18 (9–31%)	18 (9–27%)	12 (6–21%)	15 (7–23%)	12 (7–18%)	12 (6–22%)
**Nutri-Score B**						
Cereals, pasta, rice	9 (2–10%)	7 (3–9%)	12 (2–11%)	8 (2–11%)	5 (4–6%)	4 (3–6%)
Bread	6 (7–23%)	7 (3–18%)	14 (2–24%)	12 (4–20%)	10 (4–28%)	12 (2–27%)
Breakfast cereals	11 (2–15%)	8 (3–15%)	3 (9–20%)	6 (3–20%)	4 (14–36%)	5 (10–30%)
Meat	21 (20–68%)	21 (16–67%)	22 (26–73%)	22 (20–68%)	20 (18–71%)	20 (18–64%)
Fish	11 (3–11%)	13 (2–13%)	16 (3–11%)	14 (3–11%)	17 (2–16%)	15 (2–14%)
Dairy products	21 (7–38%)	21 (8–45%)	22 (5–31%)	22 (11–38%)	20 (4–29%)	20 (12–36%)
Eggs	9 (5–12%)	9 (5–13%)	8 (4–14%)	8 (4–12%)	7 (4–14%)	6 (4–12%)
**Nutri-Score C**						
Bread	21 (11–75%)	21 (12–74%)	22 (15–76%)	22 (16–74%)	20 (13–80%)	20 (9–80%)
Breakfast cereals	19 (4–26%)	18 (4–26%)	10 (3–13%)	14 (3–13%)	5 (4–11%)	8 (3–10%)
Fine bakery wares	9 (3–18%)	13 (3–20%)	8 (3–17%)	13 (3–17%)	12 (3–24%)	12 (4–25%)
Nuts and seeds	4 (3–9%)	4 (3–6%)	10 (2–11%)	7 (3–9%)	4 (2–9%)	3 (4–5%)
Meat	14 (2–28%)	12 (7–20%)	16 (2–31%)	11 (7–21%)	13 (3–17%)	11 (2–20%)
Dairy products	9 (5–26%)	11 (2–27%)	12 (2–9%)	10 (4–12%)	4 (3–11%)	5 (3–14%)
Eggs	6 (5–11%)	7 (4–11%)	11 (4–11%)	9 (5–13%)	10 (5–14%)	8 (3–13%)
Fats and oils	5 (3–10%)	4 (6–10%)	5 (4–11%)	3 (5–9%)	8 (3–28%)	7 (4–23%)
Coffee, tea, cocoa	1 (2–2%)	1 (3–3%)	4 (6–16%)	9 (4–14%)	6 (3–19%)	9 (5–20%)
**Nutri-Score D**						
Fine bakery wares	15 (8–30%)	18 (8–34%)	15 (7–20%)	18 (4–25%)	16 (6–23%)	17 (9–29%)
Meat	17 (3–27%)	17 (3–23%)	15 (7–30%)	15 (3–19%)	14 (6–29%)	12 (6–25%)
Cheese	19 (6–21%)	16 (7–18%)	18 (6–19%)	18 (8–19%)	13 (6–21%)	17 (6–21%)
Sugar	7 (6–13%)	7 (5–15%)	7 (5–11%)	10 (6–14%)	9 (3–14%)	11 (4–20%)
Fats and oils	21 (14–46%)	21 (14–48%)	22 (14–46%)	22 (15–48%)	20 (14–54%)	20 (12–54%)
Alcohol	0 (0–0%)	0 (0–0%)	19 (8–31%)	4 (6–13%)	19 (7–26%)	3 (4–17%)
Seasoning, sauces, condiments	7 (6–17%)	7 (7–18%)	7 (8–14%)	9 (6–16%)	5 (9–13%)	10 (7–17%)
**Nutri-Score E**						
Fine bakery wares	19 (7–41%)	20 (8–41%)	19 (7–32%)	21 (10–38%)	19 (7–43%)	20 (8–47%)
Meat	20 (9–34%)	20 (5–23%)	22 (8–57%)	21 (7–41%)	20 (10–48%)	17 (10–38%)
Cheese	10 (5–23%)	9 (6–24%)	12 (9–26%)	12 (8–38%)	15 (3–40%)	15 (5–55%)
Chocolate	20 (6–32%)	20 (10–32%)	17 (7–25%)	18 (8–25%)	9 (3–16%)	13 (3–16%)
Fruit juices	19 (9–54%)	19 (10–53%)	14 (5–27%)	17 (5–33%)	12 (5–21%)	17 (4–32%)
Soft drinks	11 (9–25%)	12 (7–23%)	5 (10–18%)	3 (13–22%)	2 (10–11%)	1 (15–15%)
Alcohol	0 (0–0%)	0 (0–0%)	18 (6–35%)	13 (7–18%)	19 (7–44%)	13 (7–20%)

a Values represent the number of countries reporting the consumption of a particular food group in their top five contributors with corresponding minimum and maximum contribution within brackets (e.g., for adolescent males, 19 countries reported cereals, pasta, or rice as a top five contributor of the Nutri-Score A rank, with a contribution ranging from 8 to 42%).

### Nutritional value of European diets as summarized by the FSAm-NPS-DI

**Figure 2:**
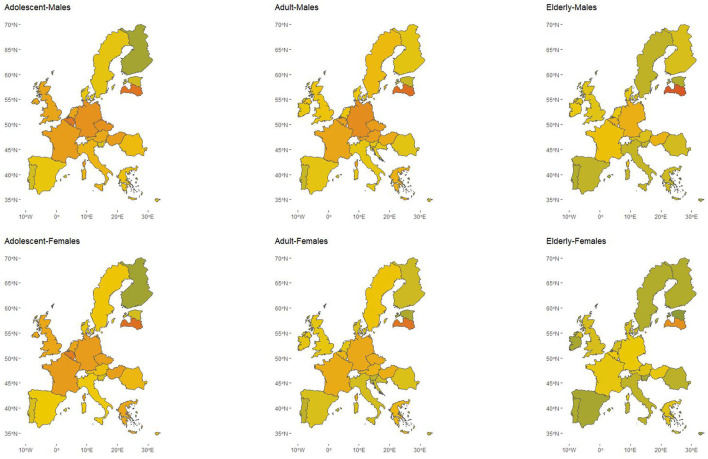
Nutritional value of diets across European populations summarized according to the Food Standard Agency modified Nutrient Profiling System Dietary Index (FSAm-NPS-DI)^1^, stratified by age-group and sex. 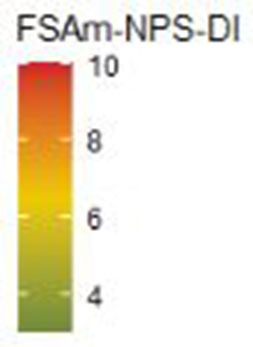 ^1^FSAm-NPS-DI is the sum of the FSAm-NPS score for each food or beverage consumed multiplied by the amount of energy provided by that product, divided by the sum of energy intake from all foods and beverages.

The median score of the FSAm-NPS-DI for diets across Europe was 6.82 (interquartile range: 6.80, 7.54) for adolescents, 6.34 (5.92, 7.19) for adults, and 5.79 (4.86, 6.36) for the elderly, indicating an overall higher nutritional value of the diets consumed by the oldest population in Europe (Supplementary Tables 1–3). Diets were of overall higher nutritional value among females, except for adolescents where no sex difference was seen (Figure 2). In general, across all age and sex groups, dietary index scores were more favorable for Estonia and Portugal, whereas the nutritional value was lower for the diets reported in Germany and Latvia.

### Time changes in the nutritional value of European diets as summarized by the FSAm-NPS-DI

**Figure 3:**
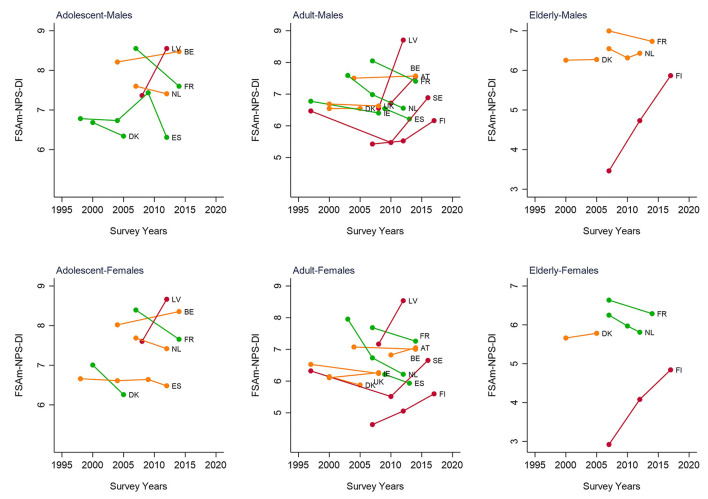
Time changes of the Food Standard Agency Nutrient Profiling System Dietary Index (FSAm-NPS-DI)^1^ in European populations, with recurrent dietary survey intake data available by EFSA, stratified by age-group and sex. AT, Austria; BE, Belgium; DK, Denmark; EN%, energy percentages; ES, Spain; FI, Finland; FR, France; LV, Latvia; IE, Ireland; NL, the Netherlands, SE, Sweden; UK, the United Kingdom. 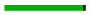 Decrease in FSAm-NPS-DI of 5% or more; 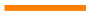 change in FSAm-NPS-DI of < 5%; 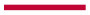 increase in FSAm-NPS-DI of 5% or more. ^1^FSAm-NPS-DI is the sum of the FSAm-NPS score for each food or beverage consumed multiplied by the amount of energy provided by that product, divided by the sum of energy intake from all foods and beverages.

Recurrent dietary surveys with the same method of dietary assessment were identified to be carried out in six countries for adolescents, namely, Belgium, Denmark, France, Latvia, the Netherlands, and Spain; in 11 countries for adults, namely, Austria, Belgium, Denmark, Finland, France, Latvia, Ireland, the Netherlands, Spain, Sweden, and the United Kingdom; and in four countries for the elderly, namely, Denmark, France, Finland, and the Netherlands. Figure 3 presents the time changes in nutritional values as summarized by the FSAm-NPS-DI for countries with available data. Both increases and decreases in the mean score of the dietary index were observed, indicating for the latter a change towards greater nutritional value, with the largest decrease (14% and 22%) for the Netherlands in adult males and females, respectively. By contrast, the dietary index score was observed to be higher in adults for Latvia (33% in males and 19% in females), Sweden (26% in males and 21% in females), Finland (12% in males and 10% in females), and Austria (13% in males only). Similarly, the largest increase in the dietary index score was observed for Latvia in adolescents and Finland in the elderly, while a decrease in the dietary index score was observed for France (in adolescent and elderly females), Denmark (in adolescents), Spain (in adolescent males), and the Netherlands (in elderly females).

## Discussion

Using the data on country-specific dietary intakes and the Nutri-Score as a benchmark for nutritional value, our study found that the nutritional value of diets varied markedly across Europe, with a dietary index ranging from 4.7 (Estonia) to 8.7 (Latvia), representing the countries with diets of better and worse nutritional values, respectively. Although a predominance of dietary items classified as A (27.6%) and D (30.0%) was observed for Europe overall, the heterogeneity of the dietary index score across countries translates also to a wide variety of A-B-C-D-E Nutri-Score profiles for individual countries. However, the foods or beverages contributing more to A were similar among the countries, in contrast to foods contributing more to the lower Nutri-Score ranks, which were a characteristic of individual countries. These observations related mostly to in-between country comparisons as only modest variations were seen by age or sex groups. Overall, healthier diets were observed for the elderly and among females (except for adolescents).

Consumers demand evidence-based information to make healthier food choices at the point of purchase and later consumption. The use of nutrition labels has the potential to inform consumers with direct and understandable messages about the nutritional value of foods and beverages they choose. As such, the Nutri-Score acts in complement to the EU-mandated nutrition declaration on prepacked food items by which energy and nutrient content have to be specified (38) to guide consumers toward informed choices and healthier options, while still accepting D- or E-classified products as part of a balanced diet, provided consumption in limited amounts or infrequently. In this regard, by visualizing the high variability in nutritional composition, the Nutri-Score provides transparency of the overall quality of foods and beverages in a relative way, allowing consumers to recognize and compare their nutritional quality and, subsequently, guide their choices toward better alternatives. Allowing a side-by-side visual comparison between the labels of similar products, either belonging to the same food group (e.g., animal or vegetable fats and oils) or among the different brands of the same product (e.g., breakfast cereals), appears to be a logical avenue to steer consumers' choices toward healthier options.

Nevertheless, with the large variability in composition across the wide range of prepackaged foods available to the consumers, there are also additional challenges in producing high-quality food consumption data feasible for use in dietary exposure assessment. Particularly, the accurate estimation of nutrient intakes would require information on brand names of foods and beverages consumed along with the availability of a complete and up-to-date food composition database that includes all available (branded) foods and beverages on the market, such as Internubel covering most products available in the Belgian market (39). In the present study, the use of a common food classification system, that is, FoodEx2, and the same food composition database, that is, NEVO, allowed for a standardized cross-country comparison of the dietary nutritional profiles as any differences exclusively originate from the nutritional value of the diet, instead of biasing the findings as a result of country-specific food composition databases. For obtaining accurate nutritional profile estimates of EU consumers' diets, a more detailed food classification system including brand names as well as brand-specific and country-specific food composition databases needs to be developed.

Our findings further underlined the region's variability in food consumption with a geographical gradient where both the Northern (Nordic) and Southern (Mediterranean) countries present diets with foods and beverages ranked higher in nutritional value, and the Central and Western European countries present diets with the lower-ranked foods and beverages. Consistent with our results, previous observations in the EPIC countries reported also a geographical gradient in nutrient intake patterns (40), with the diets of the highest nutritional value more frequently observed among the consumers of the Mediterranean region (Spain, Greece, and Italy) and of the Nordic region (Norway) (18, 19). Nevertheless, the predominance of Nutri-Score ranks A and D in all European diets relates to the similar main sources of energy intake that consisted of grains and grain-based products, fats and oils, meat, dairy products, and composite dishes. According to our data, we can deduce that within food groups, there is an enormously rich variety of foods available, hence large variability in their nutritional composition, and in particular, the grain, meat, and dairy products were often important contributors to multiple Nutri-Score ranks with varying proportions. This, however, aligns with the purpose of Nutri-Score of discriminating nutritional quality of foods and beverages among all food groups, and herewith, it was designed to maximize the distribution of products within a food group across as many Nutri-Score ranks as possible, providing nutritionally appropriate and broadening consumer choices (36, 41). Hence, this maximization of distribution was not applicable for food groups with limited variation in nutrition composition or ever high contents in one or various nutrients, as they may be concentrated in fewer Nutri-Score ranks, such as sugar confectionary, including sugars, candy, chocolate, soft drinks, and alcoholic beverages that are all in the lower Nutri-Score ranks. Therefore, food choices, which are partly formed by cultural/regional food traditions, play an important role in the nutritional value of a consumer's diet.

The Nutri-Score as a front-of-pack label is recognized as a promising strategy to encourage healthier food choices for European consumers, as shown by an online experimental study in 12 European countries (11). Controversies exist about its efficacy on actual consumers' purchasing behavior in diverse real-life settings, with complete food and beverage assortment classified according to the Nutri-Score (13, 42–44), as well as about its ability to align with other food dimensions, such as the degree of food processing, as evaluated by, for example, the NOVA classification (45, 46). While the Nutri-Score is still to be implemented in a number of European countries, its actual effectiveness would nevertheless be highly dependent on the uptake of the Nutri-Score by food retailers and manufacturers. Because of its voluntary characteristic, the uptake in Belgium was estimated to be only roughly 10% of the total supply in the first year of implementation, with the majority from retailer-branded and A- or B-classified products (47). The Nutri-Score display is expected to have continued deployment and improvement, as has been observed in France during the 3 years after its first adoption (48). As confirmed by evidence from surveys conducted in France, a growing number of foods and beverages displaying the Nutri-Score likely signifies an improved nutritional quality available for consumers' shopping baskets (49). This at least argues for the necessity of a (harmonized) mandatory front-of-pack label, as intended to be adopted by the European Commission's Farm to Fork Strategy (27), aimed at facilitating informed health-conscious food choices by all European consumers to promote nutrition equity.

Routine dietary surveys, following the same methodology of participant selection and diet collection, allow for food consumption estimates over time and hereby providing an evidence base for investigating changes in food consumption and, when repeated at regular intervals, establishing trends in such changes that might be related to the (rapidly changing) food environment and food and nutrition policies in place. Such standardized collection of accurate, harmonized, and detailed individual-level food consumption data would improve consistency and reliability of dietary estimates from the consumer domain and, when reported consistently across countries, also enable cross-country comparison of the diet, as aimed by the EFSA initiative of the European Union Menu Project launched in 2014 (50). Our study relied on available dietary data by the EFSA dietary database and hence is challenged for cross-country comparison, as mentioned in our previous publication (51). In addition, the underlying dietary surveys included and even those from most recent years are dated from several years before the implementation of the Nutri-Score, implying that with evolving food habits, the nutritional profile of a country as of today might differ from the one presented here. Particularly, an improvement in the nutritional value of the diet is expected for those countries that officially implemented or formally adopted the Nutri-Score, as anticipated by the mounting research body on the effectiveness of the Nutri-Score (5). Future investigations, using standardized methodological approaches with conducts at repeated intervals, should be undertaken to investigate the effectiveness of the Nutri-Score on food purchases and subsequent consumption in real-life settings over time and across population (sub)groups between and within countries.

In conclusion, our secondary analyses of harmonized country-specific dietary intakes aimed at describing the nutritional value of diets across Europe according to the Nutri-Score highlighted a considerable variation across countries, with modestly healthier profiles for the elderly and among females. Nevertheless, similar main food group contributors to Nutri-Score rank A and more diverse contributions for the lower Nutri-Score ranks were identified across countries and by age and sex groups, to a lesser extent.

## Data availability statement

Publicly available datasets were analyzed in this study. These data can be found here: https://data.europa.eu/data/datasets/the-efsa-comprehensive-european-food-consumption-database?locale=en; https://nevo-online.rivm.nl/. In addition, a copy of the Dutch Food Consumption Survey (FCS) 2012-2016 was requested via https://www.wateetnederland.nl/publicaties-en-datasets/datasets, and was used to initiate the linkage between FoodEx2 and food composition data, i.e. NEVO, the Dutch Food Composition Table.

## Author contributions

JP and EM conceptualized and designed the study, identified the relevant data sources, retrieved the data, and wrote the manuscript. EM performed the statistical analyses. Both authors approved the submitted version.
