# Supplementary material for: Mapping the nutritional value of diets across Europe according to the Nutri-Score front-of-pack label
Source: Front Nutr. 2023 Jan 13;9:1080858. doi: 10.3389/fnut.2022.1080858 (PMC9880413; doi:10.3389/fnut.2022.1080858)
Supplement: Supplementary file 1 [file Data_Sheet_1.docx]

Supplementary Material

# Supplementary Tables

**Supplementary Table 1** General characteristics of the most recent dietary surveys conducted in the European adolescent population, as available by EFSA, and the nutritional value of the diets according to the Nutri-Score classification, expressed in proportion of daily food consumption (grams) and in share (%) of dietary energy intake from A-, B-, C-, D- and E-classified foods and beverages, and according to the Food Standard Agency modified Nutrient Profiling System Dietary Index (FSAm-NPS-DI)^1^, ordered alphabetically and stratified by sex.

| **Country** | **Method of dietary assessment** | **Survey code** |  | | **Nutri-Score classification** | | | | | | | | | | **FSAm-NPS-DI** |
| --- | --- | --- | --- | --- | --- | --- | --- | --- | --- | --- | --- | --- | --- | --- | --- |
|  |  |  | **Year** | **Grams** | | | | | | **Energy intake** | | | | |  |
|  |  |  |  | **%A** | | **%B** | **%C** | **%D** | **%E** | **%EN A** | **%EN B** | **%EN C** | **%EN D** | **%EN E** |  |
| **EUROPE** |  |  |  |  | |  |  |  |  |  |  |  |  |  |  |
| All countries studied |  |  |  | 60.5 | | 11.6 | 8.3 | 9.8 | 9.7 | 28.0 | 12.5 | 18.1 | 27.4 | 14.0 | 6.82 |
| Males |  |  |  | 59.2 | | 11.7 | 8.6 | 10.6 | 10.0 | 27.5 | 12.7 | 18.6 | 27.2 | 13.8 | 6.85 |
| Females |  |  |  | 61.9 | | 11.5 | 8.1 | 9.1 | 9.5 | 28.4 | 12.3 | 17.6 | 27.6 | 14.1 | 6.78 |
| **MALE** |  |  |  |  | |  |  |  |  |  |  |  |  |  |  |
| Austria | 24HR | AT-ADOLESCENTS-2018-2 | 2018 | 62.1 | | 11.9 | 7.8 | 8.2 | 10.0 | 22.9 | 14.2 | 20.6 | 25.1 | 17.2 | 7.31 |
| Belgium | 24HR, FFQ | NATIONAL-FCS-2014 | 2014 | 56.2 | | 7.7 | 9.2 | 15.4 | 11.5 | 21.6 | 8.6 | 21.2 | 30.4 | 18.1 | 8.48 |
| Cyprus | 24HR | CY 2014-2017-LOT2 | 2014 | 68.0 | | 11.4 | 6.6 | 5.8 | 8.1 | 22.3 | 19.7 | 19.4 | 27.0 | 11.6 | 6.59 |
| Czech Republic | 24HR | SISP04 | 2003 | 63.1 | | 6.6 | 5.3 | 9.2 | 15.8 | 28.6 | 8.5 | 13.9 | 31.8 | 17.2 | 7.90 |
| Denmark | Food record | DANSDA 2005-08 | 2005 | 65.5 | | 7.1 | 4.7 | 11.5 | 11.2 | 37.4 | 8.1 | 13.0 | 26.1 | 15.4 | 6.34 |
| Estonia | 24HR | DIET-2014-EST-A | 2013 | 58.2 | | 18.5 | 3.9 | 9.4 | 10.0 | 35.3 | 21.0 | 9.6 | 23.9 | 10.1 | 4.63 |
| Estonia | 24HR | DIET-2014-EST-C | 2013 | 64.8 | | 10.5 | 7.2 | 7.2 | 10.3 | 38.1 | 10.4 | 13.4 | 25.7 | 12.3 | 5.73 |
| Finland | 48HR | NWSSP07_08 | 2007 | 68.7 | | 7.4 | 2.8 | 10.2 | 10.9 | 51.2 | 8.0 | 3.5 | 27.2 | 10.1 | 4.44 |
| France | 24HR, FFQ | INCA3 | 2014 | 60.4 | | 8.4 | 10.0 | 10.5 | 10.8 | 22.9 | 11.4 | 22.3 | 30.1 | 13.4 | 7.59 |
| Germany | 24HR | NATIONAL NUTRITION SURVEY II | 2007 | 43.6 | | 11.8 | 12.8 | 15.7 | 16.2 | 23.2 | 11.8 | 18.6 | 26.8 | 19.5 | 7.89 |
| Greece | 24HR | GR-EFSA-LOT2 2014-2015 | 2014 | 45.7 | | 23.9 | 10.9 | 12.6 | 6.8 | 17.1 | 20.5 | 21.3 | 30.8 | 10.3 | 6.72 |
| Hungary | 24HR, FPQ | EU MENU DIETARY SURVEY OF HUNGARY | 2018 | 67.5 | | 10.4 | 9.1 | 8.0 | 5.0 | 24.8 | 10.3 | 20.7 | 29.6 | 14.6 | 7.60 |
| Italy | Food record | INRAN SCAI 2005-06 | 2005 | 61.0 | | 10.5 | 7.9 | 14.2 | 6.4 | 27.0 | 9.6 | 15.6 | 37.7 | 10.1 | 7.02 |
| Latvia | 24HR, FPQ | LATVIA_2014 | 2012 | 56.1 | | 15.1 | 9.4 | 9.1 | 10.2 | 27.1 | 8.3 | 18.0 | 26.0 | 20.6 | 8.56 |
| The Netherlands | 24HR, LSQ | FCS2016_CORE | 2012 | 49.2 | | 8.4 | 15.5 | 14.5 | 12.4 | 27.2 | 7.5 | 24.1 | 26.3 | 14.9 | 7.40 |
| Portugal | 24HR, FPQ | IAN.AF 2015-2016 | 2015 | 66.0 | | 7.2 | 8.3 | 9.0 | 9.5 | 31.9 | 13.4 | 20.8 | 24.5 | 9.4 | 5.76 |
| Romania | 24HR | RO-DIET-NATIONAL-STUDY-2019 | 2019 | 71.2 | | 7.2 | 11.7 | 6.0 | 3.8 | 22.0 | 12.0 | 31.7 | 21.2 | 13.0 | 6.85 |
| Slovenia | 24HR, FPQ | SI.MENU-2018 | 2017 | 59.5 | | 14.6 | 9.9 | 9.5 | 6.5 | 27.7 | 17.2 | 19.6 | 22.3 | 13.3 | 5.92 |
| Spain | 24HR, FPQ | ENALIA | 2012 | 54.3 | | 18.3 | 8.7 | 9.5 | 9.2 | 20.7 | 19.6 | 20.3 | 27.9 | 11.5 | 6.31 |
| Sweden | Food record | RIKSMATEN ADOLESCENTS 2016 | 2016 | 54.3 | | 13.8 | 9.0 | 14.5 | 8.3 | 27.4 | 15.9 | 18.8 | 28.7 | 9.1 | 6.24 |
| United Kingdom | Food record | NDNS ROLLING PROGRAMME YEARS 1-3 | 2008 | 46.7 | | 14.2 | 9.5 | 12.5 | 17.1 | 21.9 | 12.8 | 23.1 | 22.8 | 19.4 | 7.40 |
| **FEMALE** |  |  |  |  | |  |  |  |  |  |  |  |  |  |  |
| Austria | 24HR | AT-ADOLESCENTS-2018-2 | 2018 | 67.6 | | 11.2 | 7.0 | 7.3 | 6.9 | 26.2 | 14.0 | 18.3 | 27.4 | 14.2 | 6.78 |
| Belgium | 24HR, FFQ | NATIONAL-FCS-2014 | 2014 | 59.0 | | 8.3 | 8.4 | 12.9 | 11.4 | 23.0 | 9.2 | 19.3 | 30.2 | 18.4 | 8.36 |
| Cyprus | 24HR | CY 2014-2017-LOT2 | 2014 | 70.3 | | 10.4 | 5.9 | 6.0 | 7.3 | 24.8 | 17.1 | 16.9 | 29.2 | 12.0 | 6.77 |
| Czech Republic | 24HR | SISP04 | 2003 | 65.9 | | 6.8 | 5.0 | 7.4 | 14.9 | 29.4 | 9.8 | 13.8 | 29.5 | 17.5 | 7.54 |
| Denmark | Food record | DANSDA 2005-08 | 2005 | 67.7 | | 7.0 | 4.4 | 9.4 | 11.5 | 37.1 | 7.6 | 14.7 | 25.7 | 15.1 | 6.26 |
| Estonia | 24HR | DIET-2014-EST-A | 2013 | 64.9 | | 12.6 | 5.7 | 6.3 | 10.3 | 39.7 | 11.4 | 14.6 | 22.6 | 11.7 | 5.15 |
| Estonia | 24HR | DIET-2014-EST-C | 2013 | 67.5 | | 11.6 | 6.2 | 6.6 | 8.1 | 37.8 | 10.5 | 13.1 | 26.1 | 12.5 | 5.77 |
| Finland | 48HR | NWSSP07_08 | 2007 | 72.4 | | 7.9 | 2.8 | 6.7 | 10.1 | 50.8 | 8.3 | 3.7 | 26.6 | 10.5 | 4.35 |
| France | 24HR, FFQ | INCA3 | 2014 | 61.4 | | 8.8 | 9.5 | 9.4 | 10.9 | 22.8 | 11.7 | 21.2 | 30.2 | 14.1 | 7.65 |
| Germany | 24HR | NATIONAL NUTRITION SURVEY II | 2007 | 50.7 | | 12.0 | 12.7 | 9.8 | 14.9 | 26.4 | 10.8 | 18.0 | 25.3 | 19.4 | 7.63 |
| Greece | 24HR | GR-EFSA-LOT2 2014-2015 | 2014 | 46.7 | | 23.5 | 8.5 | 14.6 | 6.6 | 17.0 | 18.9 | 18.2 | 35.2 | 10.6 | 7.37 |
| Hungary | 24HR, FPQ | EU MENU DIETARY SURVEY OF HUNGARY | 2018 | 66.9 | | 10.5 | 9.9 | 7.9 | 4.8 | 25.6 | 9.8 | 19.9 | 30.1 | 14.6 | 7.53 |
| Italy | Food record | INRAN SCAI 2005-06 | 2005 | 64.4 | | 11.6 | 6.5 | 12.1 | 5.4 | 28.8 | 11.5 | 13.8 | 36.8 | 9.0 | 6.53 |
| Latvia | 24HR, FPQ | LATVIA_2014 | 2012 | 57.9 | | 15.8 | 8.2 | 8.6 | 9.5 | 27.0 | 9.1 | 16.2 | 25.9 | 21.9 | 8.68 |
| The Netherlands | 24HR, LSQ | FCS2016_CORE | 2012 | 54.1 | | 8.5 | 16.2 | 10.2 | 11.0 | 28.1 | 7.0 | 23.5 | 26.1 | 15.3 | 7.41 |
| Portugal | 24HR, FPQ | IAN.AF 2015-2016 | 2015 | 66.0 | | 8.0 | 8.2 | 8.7 | 9.1 | 30.2 | 14.7 | 19.6 | 26.3 | 9.2 | 5.87 |
| Romania | 24HR | RO-DIET-NATIONAL-STUDY-2019 | 2019 | 70.9 | | 8.2 | 11.3 | 6.2 | 3.4 | 22.3 | 13.3 | 28.2 | 23.1 | 13.0 | 6.96 |
| Slovenia | 24HR, FPQ | SI.MENU-2018 | 2017 | 60.8 | | 14.4 | 9.1 | 8.6 | 7.1 | 28.9 | 17.0 | 17.3 | 22.9 | 13.8 | 5.96 |
| Spain | 24HR, FPQ | ENALIA | 2012 | 57.9 | | 16.0 | 7.9 | 9.0 | 9.2 | 22.0 | 18.5 | 18.1 | 28.6 | 12.9 | 6.48 |
| Sweden | Food record | RIKSMATEN ADOLESCENTS 2016 | 2016 | 56.2 | | 13.7 | 8.8 | 12.0 | 9.3 | 25.7 | 14.9 | 19.2 | 29.3 | 10.9 | 6.59 |
| United Kingdom | Food record | NDNS ROLLING PROGRAMME YEARS 1-3 | 2008 | 50.1 | | 14.2 | 8.1 | 10.9 | 16.7 | 23.5 | 12.5 | 21.2 | 23.3 | 19.5 | 7.37 |

Abbreviations: 24HR, 24-hour dietary recall; 48HR, 48-hour dietary recall; %EN, energy percentage, i.e. amount of kcal relative to total amount of kcal consumed; FFQ, Food Frequency Questionnaire; FPQ, Food Prosperity Questionnaire; LSQ, lifestyle questionnaire.

^1^ FSAm-NPS-DI is the sum of the FSAm-NPS score for each food or beverage consumed multiplied by the amount of energy provided by that product, divided by the sum of energy intake from all foods and beverages.

**Supplementary Table 2** General characteristics of the most recent dietary surveys conducted in the European adult population, as available by the EFSA dietary database, and the nutritional value of the diets according to the Nutri-Score classification, expressed in proportion of daily food consumption (grams) and in share (%) of dietary energy intake from A-, B-, C-, D- and E-classified foods and beverages, and according to the Food Standard Agency modified Nutrient Profiling System Dietary Index (FSAm-NPS-DI)^1^, ordered alphabetically and stratified by sex.

| **Country** | **Method of dietary assessment** | **Survey code** | **Year** | **Nutri-Score classification** | | | | | | | | | | **FSAm-NPS-DI** |
| --- | --- | --- | --- | --- | --- | --- | --- | --- | --- | --- | --- | --- | --- | --- |
|  |  |  |  | **Grams** | | | | | **Energy intake** | | | | |  |
|  |  |  |  | **%A** | **%B** | **%C** | **%D** | **%E** | **%EN A** | **%EN B** | **%EN C** | **%EN D** | **%EN E** |  |
| **EUROPE** |  |  |  |  |  |  |  |  |  |  |  |  |  |  |
| All countries studied |  |  |  | 56.4 | 11.8 | 13.3 | 12.3 | 6.2 | 27.6 | 12.9 | 17.0 | 30.0 | 12.5 | 6.34 |
| Males |  |  |  | 53.2 | 10.9 | 13.4 | 15.8 | 6.7 | 26.0 | 12.4 | 17.4 | 31.2 | 12.9 | 6.54 |
| Females |  |  |  | 59.7 | 12.7 | 13.2 | 8.8 | 5.7 | 29.0 | 13.4 | 16.6 | 28.8 | 12.2 | 6.22 |
| **MALE** |  |  |  |  |  |  |  |  |  |  |  |  |  |  |
| Austria | 24HR | AT-NATIONAL-2016 | 2014 | 56.8 | 9.5 | 13.0 | 13.2 | 7.6 | 22.5 | 11.8 | 18.4 | 31.5 | 15.8 | 7.57 |
| Belgium | 24HR, FFQ | NATIONAL-FCS-2014 | 2014 | 49.6 | 8.2 | 16.4 | 19.1 | 6.6 | 23.8 | 9.1 | 19.3 | 34.7 | 13.1 | 7.57 |
| Croatia | 24HR, 48HR | NIPNOP-HAH-2011-2012 | 2011 | 63.8 | 7.7 | 7.2 | 15.2 | 6.2 | 30.8 | 14.4 | 10.4 | 28.7 | 15.7 | 6.25 |
| Cyprus | 24HR | CY 2014-2017-LOT2 | 2014 | 65.7 | 9.9 | 8.7 | 10.3 | 5.4 | 24.1 | 21.3 | 14.2 | 30.1 | 10.3 | 5.75 |
| Czech Republic | 24HR | SISP04 | 2003 | 54.9 | 4.9 | 4.1 | 27.9 | 8.2 | 25.2 | 8.3 | 12.7 | 38.8 | 14.9 | 7.60 |
| Denmark | Food record | DANSDA 2005-08 | 2005 | 46.5 | 7.5 | 20.7 | 18.7 | 6.7 | 32.5 | 8.2 | 13.7 | 32.9 | 12.8 | 6.54 |
| Estonia | 24HR | DIET-2014-EST-A | 2013 | 56.4 | 12.1 | 13.6 | 12.0 | 5.9 | 35.4 | 13.3 | 11.5 | 29.0 | 10.7 | 5.44 |
| Finland | 24HR | FINDIET2017 | 2017 | 53.4 | 11.3 | 18.6 | 10.1 | 6.6 | 30.0 | 14.8 | 15.8 | 27.0 | 12.4 | 6.17 |
| France | 24HR, FFQ | INCA3 | 2014 | 53.5 | 8.4 | 18.4 | 12.7 | 7.1 | 22.1 | 10.1 | 22.3 | 33.1 | 12.5 | 7.40 |
| Germany | 24HR | NATIONAL NUTRITION SURVEY II | 2007 | 40.4 | 10.7 | 20.0 | 18.7 | 10.3 | 23.3 | 9.8 | 16.2 | 34.6 | 16.2 | 7.99 |
| Greece | 24HR | GR-EFSA-LOT2 2014-2015 | 2014 | 54.5 | 11.1 | 9.9 | 18.4 | 6.1 | 16.4 | 13.2 | 22.7 | 38.0 | 9.8 | 7.13 |
| Hungary | 24HR, FPQ | EU MENU DIETARY SURVEY OF HUNGARY | 2018 | 67.0 | 9.5 | 7.8 | 11.3 | 4.4 | 25.8 | 10.3 | 19.0 | 30.1 | 14.9 | 7.28 |
| Ireland | Food record | NANS 2012 | 2008 | 25.1 | 22.3 | 25.1 | 20.3 | 7.1 | 26.2 | 13.4 | 18.9 | 27.9 | 13.6 | 6.40 |
| Italy | Food record | INRAN SCAI 2005-06 | 2005 | 61.9 | 8.4 | 9.8 | 15.8 | 4.2 | 29.9 | 9.3 | 13.8 | 39.2 | 7.9 | 6.28 |
| Latvia | 24HR, FPQ | LATVIA_2014 | 2012 | 43.1 | 18.1 | 15.0 | 15.1 | 8.8 | 24.5 | 9.3 | 15.6 | 31.1 | 19.5 | 8.72 |
| The Netherlands | 24HR, LSQ | FCS2016_CORE | 2012 | 41.8 | 10.8 | 23.9 | 17.3 | 6.2 | 28.4 | 9.0 | 22.4 | 29.8 | 10.3 | 6.55 |
| Portugal | 24HR, FPQ | IAN.AF 2015-2016 | 2015 | 61.6 | 8.4 | 9.6 | 14.7 | 5.6 | 29.5 | 17.0 | 15.9 | 29.1 | 8.6 | 5.44 |
| Romania | 24HR | RO-DIET-NATIONAL-STUDY-2019 | 2019 | 65.0 | 6.7 | 13.7 | 11.0 | 3.5 | 23.5 | 12.1 | 29.3 | 22.7 | 12.4 | 6.14 |
| Slovenia | 24HR, FPQ | SI.MENU-2018 | 2017 | 59.0 | 9.8 | 11.3 | 14.2 | 5.7 | 28.3 | 13.9 | 17.3 | 27.3 | 13.2 | 6.25 |
| Spain | 24HR | ENALIA2 | 2013 | 58.2 | 12.6 | 8.9 | 14.4 | 5.9 | 22.6 | 18.3 | 16.8 | 31.8 | 10.6 | 6.22 |
| Sweden | Food record | RIKSMATEN 2016 | 2016 | 47.9 | 13.4 | 12.5 | 18.2 | 8.0 | 23.6 | 14.4 | 19.2 | 31.0 | 11.7 | 6.89 |
| United Kingdom | Food record | NDNS ROLLING PROGRAMME YEARS 1-3 | 2008 | 43.8 | 19.8 | 7.4 | 19.5 | 9.4 | 24.5 | 12.6 | 18.4 | 27.9 | 16.5 | 6.66 |
| **FEMALE** |  |  |  |  |  |  |  |  |  |  |  |  |  |  |
| Austria | 24HR | AT-NATIONAL-2016 | 2014 | 59.4 | 10.9 | 16.7 | 7.0 | 6.0 | 25.6 | 12.4 | 17.6 | 29.7 | 14.6 | 7.06 |
| Belgium | 24HR, FFQ | NATIONAL-FCS-2014 | 2014 | 57.2 | 10.4 | 16.6 | 9.6 | 6.2 | 26.8 | 10.9 | 18.8 | 30.7 | 12.8 | 7.01 |
| Croatia | 24HR, 48HR | NIPNOP-HAH-2011-2012 | 2011 | 72.7 | 7.1 | 7.9 | 7.5 | 4.8 | 34.9 | 15.1 | 10.1 | 27.0 | 12.9 | 5.57 |
| Cyprus | 24HR | CY 2014-2017-LOT2 | 2014 | 71.8 | 8.9 | 8.9 | 5.5 | 4.9 | 28.4 | 19.1 | 15.2 | 27.6 | 9.7 | 5.57 |
| Czech Republic | 24HR | SISP04 | 2003 | 73.4 | 5.8 | 3.6 | 9.6 | 7.6 | 28.4 | 10.8 | 12.9 | 34.0 | 14.0 | 7.28 |
| Denmark | Food record | DANSDA 2005-08 | 2005 | 58.8 | 9.4 | 14.8 | 10.2 | 6.9 | 36.7 | 8.6 | 13.4 | 28.2 | 13.1 | 5.87 |
| Estonia | 24HR | DIET-2014-EST-A | 2013 | 60.4 | 14.2 | 15.5 | 5.5 | 4.4 | 39.6 | 14.6 | 11.1 | 25.1 | 9.5 | 4.70 |
| Finland | 24HR | FINDIET2017 | 2017 | 59.9 | 12.5 | 16.2 | 6.1 | 5.3 | 30.9 | 16.8 | 16.4 | 24.7 | 11.1 | 5.60 |
| France | 24HR, FFQ | INCA3 | 2014 | 55.1 | 12.3 | 17.8 | 8.8 | 5.9 | 23.4 | 11.3 | 20.1 | 33.8 | 11.5 | 7.25 |
| Germany | 24HR | NATIONAL NUTRITION SURVEY II | 2007 | 48.1 | 12.4 | 21.1 | 9.4 | 9.0 | 27.6 | 10.4 | 16.6 | 29.8 | 15.6 | 7.34 |
| Greece | 24HR | GR-EFSA-LOT2 2014-2015 | 2014 | 60.7 | 13.7 | 7.2 | 13.4 | 5.0 | 19.7 | 15.6 | 17.5 | 37.0 | 10.2 | 6.97 |
| Hungary | 24HR, FPQ | EU MENU DIETARY SURVEY OF HUNGARY | 2018 | 71.5 | 11.9 | 6.9 | 6.4 | 3.4 | 28.8 | 10.7 | 16.6 | 29.1 | 14.9 | 7.17 |
| Ireland | Food record | NANS 2012 | 2008 | 29.2 | 27.0 | 26.9 | 9.6 | 7.3 | 28.7 | 14.0 | 18.9 | 23.6 | 14.9 | 6.24 |
| Italy | Food record | INRAN SCAI 2005-06 | 2005 | 67.5 | 9.8 | 8.6 | 10.9 | 3.2 | 32.5 | 10.8 | 12.2 | 37.9 | 6.6 | 5.79 |
| Latvia | 24HR, FPQ | LATVIA_2014 | 2012 | 48.9 | 17.3 | 18.0 | 9.3 | 6.4 | 26.7 | 10.1 | 15.9 | 27.0 | 20.3 | 8.55 |
| The Netherlands | 24HR, LSQ | FCS2016_CORE | 2012 | 47.1 | 14.7 | 23.8 | 8.9 | 5.5 | 30.3 | 9.9 | 23.1 | 26.2 | 10.5 | 6.21 |
| Portugal | 24HR, FPQ | IAN.AF 2015-2016 | 2015 | 67.8 | 9.6 | 11.0 | 7.4 | 4.3 | 33.7 | 16.3 | 15.8 | 26.0 | 8.3 | 5.17 |
| Romania | 24HR | RO-DIET-NATIONAL-STUDY-2019 | 2019 | 71.5 | 7.4 | 12.6 | 6.0 | 2.5 | 26.7 | 14.6 | 24.2 | 23.1 | 11.5 | 5.99 |
| Slovenia | 24HR, FPQ | SI.MENU-2018 | 2017 | 66.0 | 12.1 | 11.2 | 7.3 | 3.5 | 31.7 | 17.1 | 16.1 | 24.7 | 10.4 | 5.26 |
| Spain | 24HR | ENALIA2 | 2013 | 64.0 | 11.9 | 8.6 | 10.3 | 5.1 | 25.6 | 18.0 | 15.1 | 32.2 | 9.1 | 5.93 |
| Sweden | Food record | RIKSMATEN 2012 | 2012 | 50.6 | 15.1 | 9.1 | 16.0 | 9.2 | 23.4 | 14.2 | 19.7 | 32.5 | 10.2 | 6.65 |
| United Kingdom | Food record | NDNS ROLLING PROGRAMME YEARS 1-3 | 2008 | 50.8 | 24.7 | 6.7 | 8.7 | 9.1 | 28.3 | 14.1 | 17.4 | 23.4 | 16.9 | 6.28 |

Abbreviations: 24HR, 24-hour dietary recall; 48HR, 48-hour dietary recall; %EN, energy percentage, i.e. amount of kcal relative to total amount of kcal consumed; FFQ, Food Frequency Questionnaire; FPQ, Food Prosperity Questionnaire; LSQ, lifestyle questionnaire.

^1^ FSAm-NPS-DI is the sum of the FSAm-NPS score for each food or beverage consumed multiplied by the amount of energy provided by that product, divided by the sum of energy intake from all foods and beverages.

**Supplementary Table 3** General characteristics of the most recent dietary surveys conducted in the European elderly population, as available by the EFSA dietary database, and the nutritional value of the diets according to the Nutri-Score classification, expressed in proportion of daily food consumption (grams) and in share (%) of dietary energy intake from A-, B-, C-, D- and E-classified foods and beverages, and according to the Food Standard Agency modified Nutrient Profiling System Dietary Index (FSAm-NPS-DI)^1^, ordered alphabetically and stratified by sex.

| **Country** | **Method of dietary assessment** | **Survey code** | **Year** | **Nutri-Score classification** | | | | | | | | | | **FSAm-NPS-DI** |
| --- | --- | --- | --- | --- | --- | --- | --- | --- | --- | --- | --- | --- | --- | --- |
|  |  |  |  | **Grams** | | | | | **Energy intake** | | | | |  |
|  |  |  |  | **%A** | **%B** | **%C** | **%D** | **%E** | **%EN A** | **%EN B** | **%EN C** | **%EN D** | **%EN E** |  |
| **EUROPE** |  |  |  |  |  |  |  |  |  |  |  |  |  |  |
| All countries studied |  |  |  | 55.7 | 13.9 | 15.3 | 10.4 | 4.7 | 31.6 | 13.1 | 16.9 | 28.0 | 10.4 | 5.79 |
| Males |  |  |  | 52.4 | 13.6 | 15.3 | 13.5 | 5.2 | 30.0 | 12.4 | 17.1 | 29.6 | 11.0 | 5.90 |
| Females |  |  |  | 58.9 | 14.2 | 15.3 | 7.4 | 4.2 | 33.3 | 13.8 | 16.7 | 26.4 | 9.7 | 5.50 |
| **MALE** |  |  |  |  |  |  |  |  |  |  |  |  |  |  |
| Austria | 24HR | ASNS - ADULTS | 2010 | 48.1 | 18.4 | 13.7 | 13.8 | 6.0 | 33.9 | 15.7 | 11.4 | 23.9 | 15.1 | 5.94 |
| Belgium | 24HR, FFQ | DIET NATIONAL 2004 | 2004 | 41.3 | 11.4 | 23.8 | 18.3 | 5.2 | 26.9 | 10.5 | 19.5 | 32.3 | 10.8 | 7.05 |
| Cyprus | 24HR | CY 2014-2017-LOT2 | 2014 | 72.7 | 8.6 | 7.8 | 7.7 | 3.3 | 29.9 | 16.4 | 17.2 | 28.4 | 8.1 | 4.82 |
| Denmark | Food record | DANSDA 2005-08 | 2005 | 44.7 | 8.6 | 22.7 | 18.7 | 5.4 | 33.5 | 9.0 | 12.8 | 33.5 | 11.1 | 6.27 |
| Estonia | 24HR | DIET-2014-EST-A | 2013 | 55.0 | 14.4 | 16.3 | 9.6 | 4.7 | 38.4 | 13.8 | 11.8 | 27.0 | 8.9 | 4.85 |
| Finland | 24HR | FINDIET2017 | 2017 | 50.4 | 12.4 | 20.3 | 10.6 | 6.3 | 31.7 | 14.4 | 16.6 | 26.2 | 11.2 | 5.87 |
| France | 24HR, FFQ | INCA3 | 2014 | 52.4 | 8.0 | 21.1 | 12.8 | 5.7 | 23.6 | 8.9 | 25.2 | 32.9 | 9.4 | 6.72 |
| Germany | 24HR | NATIONAL NUTRITION SURVEY II | 2007 | 41.3 | 13.3 | 21.6 | 16.9 | 6.9 | 29.2 | 8.8 | 13.9 | 36.3 | 11.7 | 7.16 |
| Greece | 24HR | GR-EFSA-LOT2 2014-2015 | 2014 | 61.6 | 12.3 | 8.5 | 13.3 | 4.3 | 25.5 | 13.4 | 20.2 | 32.9 | 8.0 | 5.61 |
| Hungary | 24HR, FPQ | EU MENU DIETARY SURVEY OF HUNGARY | 2018 | 66.0 | 11.7 | 6.8 | 10.8 | 4.7 | 28.5 | 8.9 | 17.3 | 31.1 | 14.2 | 7.06 |
| Ireland | Food record | NANS 2012 | 2008 | 26.3 | 32.8 | 19.2 | 16.9 | 4.9 | 28.7 | 12.3 | 16.6 | 31.2 | 11.2 | 6.42 |
| Italy | Food record | INRAN SCAI 2005-06 | 2005 | 64.0 | 9.3 | 9.3 | 14.1 | 3.3 | 32.4 | 10.2 | 13.9 | 37.2 | 6.2 | 5.38 |
| Latvia | 24HR, FPQ | LATVIA_2014 | 2012 | 47.2 | 16.1 | 13.2 | 16.4 | 7.2 | 25.0 | 8.6 | 14.2 | 31.5 | 20.7 | 9.16 |
| The Netherlands | 24HR, LSQ | FCS2016_CORE | 2012 | 39.7 | 13.3 | 27.4 | 13.8 | 5.8 | 29.2 | 9.8 | 21.3 | 28.8 | 10.8 | 6.42 |
| Portugal | 24HR, FPQ | IAN.AF 2015-2016 | 2015 | 64.0 | 7.4 | 9.7 | 15.0 | 3.9 | 33.2 | 15.9 | 14.4 | 29.1 | 7.5 | 4.86 |
| Romania | 24HR | RO-DIET-NATIONAL-STUDY-2019 | 2019 | 66.6 | 6.8 | 14.8 | 8.6 | 3.2 | 28.6 | 11.6 | 25.0 | 23.6 | 11.1 | 5.71 |
| Slovenia | 24HR, FPQ | SI.MENU-2018 | 2017 | 56.9 | 11.5 | 13.1 | 11.6 | 6.9 | 34.7 | 14.3 | 13.1 | 25.2 | 12.7 | 5.37 |
| Spain | 24HR | ENALIA2 | 2013 | 63.0 | 10.8 | 8.6 | 12.7 | 4.9 | 27.2 | 15.9 | 18.1 | 31.1 | 7.7 | 5.26 |
| Sweden | Food record | RIKSMATEN 2010 | 2010 | 44.5 | 15.4 | 20.9 | 12.9 | 6.2 | 32.6 | 15.5 | 19.7 | 22.4 | 9.7 | 5.23 |
| United Kingdom | Food record | NDNS ROLLING PROGRAMME YEARS 1-3 | 2008 | 43.1 | 29.5 | 7.1 | 14.7 | 5.7 | 27.0 | 14.0 | 18.7 | 26.6 | 13.7 | 6.14 |
| **FEMALE** |  |  |  |  |  |  |  |  |  |  |  |  |  |  |
| Austria | 24HR | ASNS - ADULTS | 2010 | 49.8 | 18.5 | 14.0 | 11.6 | 6.1 | 30.4 | 16.3 | 14.7 | 26.0 | 12.5 | 6.12 |
| Belgium | 24HR, FFQ | DIET NATIONAL 2004 | 2004 | 49.9 | 12.9 | 23.9 | 9.6 | 3.8 | 28.3 | 14.1 | 18.9 | 29.3 | 9.4 | 6.52 |
| Cyprus | 24HR | CY 2014-2017-LOT2 | 2014 | 76.1 | 7.8 | 8.6 | 5.0 | 2.5 | 35.8 | 15.2 | 15.0 | 28.3 | 5.7 | 4.25 |
| Denmark | Food record | DANSDA 2005-08 | 2005 | 54.0 | 10.2 | 19.6 | 11.2 | 5.0 | 37.0 | 9.3 | 13.0 | 28.5 | 12.2 | 5.78 |
| Estonia | 24HR | DIET-2014-EST-A | 2013 | 62.2 | 13.8 | 16.0 | 4.6 | 3.3 | 43.3 | 15.1 | 11.4 | 23.3 | 7.0 | 3.87 |
| Finland | 24HR | FINDIET2017 | 2017 | 57.3 | 13.7 | 18.9 | 5.7 | 4.5 | 33.5 | 17.5 | 18.7 | 22.3 | 8.0 | 4.84 |
| France | 24HR, FFQ | INCA3 | 2014 | 56.4 | 14.2 | 18.3 | 7.1 | 3.9 | 26.5 | 12.0 | 20.8 | 32.0 | 8.7 | 6.28 |
| Germany | 24HR | NATIONAL NUTRITION SURVEY II | 2007 | 49.4 | 13.1 | 23.1 | 7.6 | 6.8 | 33.8 | 9.8 | 14.6 | 30.3 | 11.5 | 6.43 |
| Greece | 24HR | GR-EFSA-LOT2 2014-2015 | 2014 | 68.1 | 11.0 | 7.2 | 10.6 | 3.2 | 26.7 | 13.1 | 16.9 | 34.5 | 8.9 | 6.10 |
| Hungary | 24HR, FPQ | EU MENU DIETARY SURVEY OF HUNGARY | 2018 | 75.6 | 10.4 | 6.6 | 5.0 | 2.4 | 32.2 | 10.8 | 16.8 | 28.2 | 11.9 | 6.35 |
| Ireland | Food record | NANS 2012 | 2008 | 33.4 | 33.5 | 25.0 | 4.3 | 3.9 | 36.6 | 16.5 | 18.7 | 19.5 | 8.7 | 4.51 |
| Italy | Food record | INRAN SCAI 2005-06 | 2005 | 69.2 | 10.2 | 8.0 | 9.9 | 2.6 | 33.3 | 11.5 | 13.0 | 36.8 | 5.5 | 5.23 |
| Latvia | 24HR, FPQ | LATVIA_2014 | 2012 | 51.9 | 15.8 | 16.0 | 10.3 | 6.0 | 30.4 | 10.6 | 14.6 | 26.7 | 17.8 | 7.91 |
| The Netherlands | 24HR, LSQ | FCS2016_CORE | 2012 | 45.5 | 16.9 | 25.4 | 7.0 | 5.1 | 32.8 | 10.8 | 21.4 | 25.2 | 9.8 | 5.80 |
| Portugal | 24HR, FPQ | IAN.AF 2015-2016 | 2015 | 71.1 | 8.8 | 11.3 | 5.9 | 2.9 | 37.2 | 15.5 | 14.9 | 23.8 | 8.7 | 4.75 |
| Romania | 24HR | RO-DIET-NATIONAL-STUDY-2019 | 2019 | 71.5 | 7.5 | 13.7 | 5.1 | 2.2 | 31.1 | 14.0 | 23.9 | 22.1 | 8.8 | 5.10 |
| Slovenia | 24HR, FPQ | SI.MENU-2018 | 2017 | 65.8 | 11.3 | 13.0 | 6.1 | 3.8 | 38.2 | 15.3 | 14.2 | 23.4 | 8.9 | 4.47 |
| Spain | 24HR | ENALIA2 | 2013 | 70.3 | 10.9 | 8.0 | 7.5 | 3.3 | 33.1 | 17.0 | 15.0 | 28.9 | 6.0 | 4.52 |
| Sweden | Food record | RIKSMATEN 2010 | 2010 | 49.3 | 14.1 | 21.8 | 8.7 | 6.1 | 33.4 | 16.2 | 20.6 | 20.2 | 9.6 | 4.84 |
| United Kingdom | Food record | NDNS ROLLING PROGRAMME YEARS 1-3 | 2008 | 52.1 | 29.5 | 6.7 | 5.5 | 6.2 | 31.7 | 14.8 | 17.2 | 21.1 | 15.2 | 5.84 |

Abbreviations: 24HR, 24-hour dietary recall; 48HR, 48-hour dietary recall; %EN, energy percentage, i.e. amount of kcal relative to total amount of kcal consumed; FFQ, Food Frequency Questionnaire; FPQ, Food Prosperity Questionnaire; LSQ, lifestyle questionnaire.

^1^ FSAm-NPS-DI is the sum of the FSAm-NPS score for each food or beverage consumed multiplied by the amount of energy provided by that product, divided by the sum of energy intake from all foods and beverages.
